# Relationships between Vitamin D_3_ and Metabolic Syndrome

**DOI:** 10.3390/ijerph16020175

**Published:** 2019-01-09

**Authors:** Sylwia Wieder-Huszla, Anna Jurczak, Małgorzata Szkup, Katarzyna Barczak, Barbara Dołęgowska, Daria Schneider-Matyka, Joanna Owsianowska, Elżbieta Grochans

**Affiliations:** 1 Department of Clinical Nursing, Pomeranian Medical University in Szczecin, 70-204 Szczecin, Poland; sylwia.huszla@pum.edu.pl (S.W.-H.); anna.jurczak@pum.edu.pl (A.J.); jowsian@pum.edu.pl (J.O.); 2 Department of Nursing, Pomeranian Medical University in Szczecin, 70-204 Szczecin, Poland; malgorzata.szkup@pum.edu.pl (M.S.); daria.schneider-matyka@pum.edu.pl (D.S.-M.); 3 Department of Conservative Dentistry and Endodontics, Pomeranian Medical University in Szczecin, 70-204 Szczecin, Poland; zstzach@pum.edu.pl; 4 Department of Microbiology, Immunology and Laboratory Medicine, Pomeranian Medical University in Szczecin, 70-204 Szczecin, Poland; barbara.dolegowska@pum.edu.pl

**Keywords:** metabolic syndrome, vitamin D_3_, women

## Abstract

The growing number of overweight and obese individuals is an alarming global problem; these conditions are risk factors for the development of health problems such as metabolic syndrome (MetS), type-2 diabetes, atherosclerosis, and cardiovascular disease. Numerous studies have suggested that vitamin D_3_ deficiency plays a role in the pathogenesis of MetS. The aim of this study was to analyze the relationship between MetS and vitamin D_3_ levels in women. Laboratory analysis demonstrated that only 26.89% of the participants had vitamin D_3_ levels close to normal, and waist-to-hip ratio (WHR) measurements revealed android obesity in 75.63% of the women. The menstruating women more often suffered from vitamin D_3_ deficiency, and less often had elevated vitamin D_3_ levels. The conclusions are as follows: (1) There were no statistically significant relationships between vitamin D_3_ levels and MetS parameters, namely the level of triglycerides, the levels of low- and high-density lipoproteins (LDL and HDL), the level of total cholesterol, and systolic and diastolic blood pressure (SBP and DBP). Vitamin D deficiency was only observed in the women with abdominal obesity. (2) Low vitamin D_3_ levels were typical of perimenopausal women. Age was a variable correlating with vitamin D. (3) The presence of menstrual cycles was an important contributor to vitamin D levels. Vitamin D deficiency was significantly more common in the menstruating women.

## 1. Introduction

Numerous reports have shown that the number of overweight and obese people has increased in recent years. The problem affects children, adolescents, and adults [1,2,3,4,5,6]. Obesity leads to many systemic disorders, including metabolic syndrome (MetS), type-2 diabetes, atherosclerosis, cardiovascular complications, cancer diseases, etc. [7,8,9]. There are reports suggesting that obesity leads to chronic inflammation, which disturbs the proper functioning of the immune and metabolic systems [10,11,12]. Increased inflammatory activation is characterized by elevated serum levels of C-reactive protein (CRP) and proinflammatory cytokines, such as interleukin-6 (IL-6), and tumor necrosis factor alpha (TNF-α) [11,12,13,14].

Over recent years, "metabolic syndrome" has become one of the most commonly used terms in the field of health sciences. The definition of MetS indicates that the development of atherosclerotic cardiovascular disease and type-2 diabetes is underlaid by interrelated risk factors [15,16]. The most important element of the etiology of MetS is insulin resistance accompanied by compensatory hyperinsulinemia, while the most-often diagnosed components are impaired glucose tolerance or diabetes, android obesity, atherogenic dyslipidemia, low levels of high-density lipoprotein (HDL) cholesterol, and increased blood pressure [16,17]. Of key importance for the development of MetS is an excess of white adipose tissue and its distribution in the visceral area. Android obesity, diagnosed on the basis of waist circumference, is thus a vital diagnostic criterion for MetS [18,19]. White adipose tissue thus plays a double role, bothfunctional and mechanical (it serves as stroma and thermal insulation protection for internal organs). It is also an active buffer for lipids, and an important endocrine organ [20,21]. Many authors have demonstrated a significant association between android obesity and the major components of MetS, namely insulin resistance, hypertension, atherogenic dyslipidemia, and hyperglycemia [22,23]. The mechanism of these relationships is very complex and not fully elucidated. Nevertheless, the epidemic of android obesity is the leading cause of the increase in the worldwide incidence of MetS [22,23].

The lack of an unambiguous view of the etiology of MetS translates into diagnostic difficulties in clinical practice. Hence, a long-lasting discussion in academic circles on MetS, its definition, and criteria is of highest diagnostic and prognostic value. Several different definitions of MetS were developed at the turn of the 21st century. In 2005, the International Diabetes Federation (IDF) established diagnostic criteria for MetS, which in fact were a modified version of the guidelines previously proposed by the experts of the National Cholesterol Education Program Adult Treatment Panel III (NCEP-ATP III). A precondition for the diagnosis of MetS was central obesity, accompanied by two out of four factors mentioned in the definition suggested by the NCEP-ATP III. However, it was not until 2009 that the IDF, with the approval of the American Heart Association/National Heart, Lung, and Blood Institute (AHA/NHLBI), offered one single definition and uniform guidelines on MetS [18,24].

MetS is thus an important field of interest and investigation for public health. Atherosclerotic cardiovascular disease remains the main cause of complications and deaths.

Vitamin D_3_ is obtained from the diet or through synthesis in the skin with the participation of ultraviolet B radiation (UVB). Next, it is metabolized to its active form, 1.25-dihydroxyvitamin D, with the aid of enzymes. There are two forms of vitamin D: vitamin D_2_ (ergocalciferol), which is found in plants and fungi, and vitamin D_3_ (cholecalciferol), which is produced by animals. In humans, solar radiation converts provitamin D_3_ into previtamin D_3_, which is then spontaneously heat-isomerized to vitamin D_3_ [25,26]. The main circulating metabolite of vitamin D is vitamin 25(OH)D, which accurately reflects the amount of vitamin D in the body, whether it originated from food or was synthesized in the skin. According to many authors, vitamin D deficiency is a worldwide phenomenon that may affect as many as 30–50% of the adult population [27,28,29]. Those especially prone to vitamin D deficiency are elderly people, due to their decreased ability to synthesize this vitamin in the skin [29].

Until recently, vitamin 25(OH)D was regarded as a factor regulating calcium phosphate and bone tissue metabolism. Reports from recent years have confirmed that it is involved in maintaining homeostasis in many tissues [30,31], and its deficiency is associated with musculoskeletal and cardiovascular disorders, as well as autoimmune, dermatological, and cancer diseases [32,33,34,35]. Furthermore, serum vitamin D levels appear to be related to obesity in both healthy and ill individuals [36,37,38,39,40,41]. The reduced bioavailability of vitamin D is observed in overweight and obese individuals, which probably results from the increased sequestration of this vitamin in adipose tissue [14,42]. Vitamin D deficiency is closely related to visceral obesity [43], which is confirmed by elevated serum levels of proinflammatory cytokines and TNF-α in overweight and obese people [12]. Taking into account the global increase in obesity, the analysis of the relationships between vitamin D levels, adipose tissue metabolism, and the development of MetS is a necessity. Our attempts to identify the determinants of MetS will undoubtedly contribute to the state of the art in the field, and enable successful therapeutic interventions.

## 2. Aim of the Study

The aim of this study was to determine the connection between vitamin D_3_ and MetS in perimenopausal women.

## 3. Material and Methods

The study involved 119 women from West Pomeranian province, Poland. The criteria for inclusion in the study were female sex, age, a diagnosis of MetS, voluntary written consent to take part in the study, and place of residence in West Pomeranian province. The exclusion criteria were oncological and psychiatric disorders and D_3_ vitamin supplementation. The patients received written information about the purpose and course of the study, and were assured that they were free to withdraw at any stage without giving a reason. The research procedure was divided into three stages, including structured medical history, anthropometric and blood pressure measurements, and serum biochemical analysis.

The patients were diagnosed with MetS if they had at least 3 out of 5 of its components, in accordance with the modified criteria proposed by the IDF in 2009. These components were waist circumference ≥80 cm, fasting glycemia ≥100 mg/dL or pharmacotherapy for hyperglycemia, triglyceride level ≥150 mg/dL or related pharmacotherapy, HDL cholesterol level <50 mg/dL or related pharmacotherapy, and elevated blood pressure (sBP ≥130 and/or dBP ≥85 mmHg) or pharmacotherapy for hypertension [27].

Anthropometric measurements were performed using a digital scale with a height rod. The patients were measured on an empty stomach, after urination, wearing light clothes and no shoes. Waist and hip measurements were taken in a standing position. Waist circumference was measured between the lower rib margin and the upper margin of the iliac crest at the end of a gentle exhalation. Hip circumference was measured at the level of the maximum protrusion of the gluteal muscles. Waist circumference ≤80 cm was regarded as normal. Based on the data obtained, we calculated the body mass index (BMI) and waist-to-hip ratio (WHR). A BMI in the range of 18.5–24.9 kg/m^2^ was regarded as normal, a BMI between 25.0 kg/m^2^ and 29.9 kg/m^2^ denoted overweight, and a BMI ≥ 30 kg/m^2^ indicated obesity. WHR was calculated by dividing waist circumference by hip circumference. Values <0.8 indicated the gynoid type of adipose tissue distribution, and values ≥0.8 indicated android type. Blood pressure, both systolic (sBP) and diastolic (dBP), was gauged in a sitting position using a manual manometer. The manometer’s cuff was wrapped snugly around the patient’s right upper arm at the heart level. The patient’s arm was supported so as to allow her to rest in that position for at least 5 min before the measurement. The cuff was adjusted for the arm circumference.

At the next stage, venous blood samples were taken on an empty stomach (minimum 8 h from the last meal) from each patient using the Vacutainer system, in compliance with the relevant regulations. The levels of the following biochemical parameters were determined in blood serum: total cholesterol (TCh, normal level: 115–190 mg/dL), HDL (normal level >50 mg/dL), low-density lipoprotein (LDL, normal level <115 mg/dL), triglycerides (TG, normal level <150 mg/dL), and vitamin D_3_ (normal level: 30–80 ng/mL or 75–200 nmol/L).

All subjects were informed in detail of the range and purpose of the study, and gave their written consent for their participation. The protocol was approved by the Bioethical Commission of the Pomeranian Medical University in Szczecin (approval number KB-0012/181/13).

### Statistical Analysis

Statistical analysis was performed in the R software environment (version 3.5.0). Descriptive statistics—such as the number of valid cases, arithmetic mean, standard deviation, and median, as well as minimum and maximum values—were applied. We also used the structure ratio and mathematical statistics, such as distribution fitting tests, nonparametric correlations, and significance-of-difference tests. Statistical significance was assumed at *p* ≤ 0.05, and high statistical significance at *p* ≤ 0.01.

## 4. Results

The mean age of the participants was 52.73 ± 7.92 years. The majority of them had completed tertiary (39.50%) and secondary (36.13%) education, with 57.98% of the women living in large conurbations. The vast majority of the women were married (75.63%) and employed (73.95%).

Excessive body weight was a major problem: 33.61% were overweight, 26.05% were obese, and 18.48% had first- or second-degree obesity. A high BMI was observed in 78.15% (93) of cases. The mean BMI of the overweight women fell within the range of 32.39 ± 29.81, and was close to the mean BMI of their obese counterparts, 32.29 ± 24.75. The values for the women with first and second-degree obesity were slightly higher (39.01 ± 30.31). Only 26.89% (32) of the participants had normal vitamin D_3_ levels, 52.94% (63) had vitamin D_3_ deficiency, and 20.16% (24) had elevated levels of the vitamin. There were no statistically significant relationships between the women’s BMIs and their vitamin D_3_ levels (*p* = 0.092; *p* = 0.291), see Table 1.

The measurement of adipose tissue distribution (WHR) showed that the vast majority of the women (75.63%) had android obesity, and almost half (48.89%) of these had low vitamin D_3_ levels. In the group without android obesity, vitamin deficiency was observed in 65.52% of the women. The analysis did not demonstrate a statistically significant relationship between the women’s waist circumference and their vitamin D_3_ levels (*p* = 0.067; *p* = 0.291), see Table 2.

No statistically significant relationships (*p* > 0.05) were found between the selected metabolic parameters (total cholesterol, LDL and the HDL fractions, and triglycerides), systolic (sBP) and diastolic blood pressure (dBP), and vitamin D_3_ levels (Table 3).

The study demonstrated a statistically significant positive correlation between the women’s age and their vitamin D_3_ levels (*p* < 0.001), see Table 4.

Age statistically contributed significantly both to the risk of vitamin D_3_ deficiency (*p* < 0.006)—vitamin D_3_ levels decreased by 6.7% (odds ratio = 0.933) with each subsequent year—and to the risk of elevated vitamin D_3_ levels (*p* < 0.002)—vitamin D_3_ levels rose by 13.1% (odds ratio = 1.131) with each subsequent year (Table 5).

Menstruating women made up 36.13% of the study sample (56.30% not menstruating). The research revealed a statistically significant relationship (*p* = 0.015; *p* = 0.01) between vitamin D_3_ levels and the menstrual cycle. The menstruating women more often had vitamin D_3_ deficiencies, and less often showed elevated vitamin D_3_ levels (Table 6).

## 5. Discussion

Advanced clinical and epidemiological studies have provided data on the factors that contribute to inadequate vitamin D_3_ levels and the diseases this can cause in humans. Research results suggest that vitamin D_3_ deficiency entails a higher incidence of autoimmune, cardiovascular, and cancer diseases [30,31,32,33,35]. Vitamin D_3_ synthesis in human skin is one factor that determines the amount of cholecalciferol available to healthy individuals. Many elements, including air pollution, lifestyle, the use of sun filters, and the dose of ultraviolet (UV) radiation received affect this synthesis [30]. At latitudes corresponding to Poland, optimal vitamin D supply is achieved from March/April to September, provided that 18% of the naked body is exposed to the sun for 15 min a day. During fall and winter, oral supplementation is recommended [30,44]. According to estimates, about 30–80% of children and adults worldwide suffer from severe vitamin D deficiency [45], which has serious consequences on health, and thus contributes to a global public health crisis [33].

Many researchers point to vitamin D_3_ deficiency as a factor in the pathogenesis of hypertension (vitamin D_3_ inhibits renin and endothelin synthesis and the proliferation of smooth muscle cells), MetS, and diabetes (development of insulin resistance). It has hence been suggested that vitamin D_3_ deficiency raises the risk of cardiovascular disease [26,27,46,47,48,49,50]. According to expert guidelines proposed in 2009, the pleiotropic effects of vitamin D_3_ can be achieved by maintaining vitamin levels in the range of 30–80 ng/mL (75–200 nm/L) in adults [44]. Vitamin D_3_ deficiency, defined as serum levels of calcifediol (or 25(OH)D) below 20 ng/mL, is observed in over half of the Polish population, with this percentage increasing to over 70% in the winter months [25,51,52,53]. Our study has demonstrated low serum vitamin 25(OH)D levels (below 30 ng/mL) in 52.94% of its participants, while the optimal (recommended) levels were only found in 26.89%.

The relationship between obesity and vitamin D_3_ has not yet been fully explicated. However, the available findings show that overweight and obese people have low serum levels of this vitamin [53,54,55,56,57]. In obese individuals, vitamin D_3_ bioavailability is limited due to its sequestration in adipose tissue [42,57,58]. Many researchers indicate that serum vitamin 25(OH)D levels are associated with obesity in both healthy and unhealthy populations [36,37,38,39,59,60,61]. Nonetheless, this relationship has not been confirmed by all authors [37,62], which corresponds with our outcomes showing no association between BMI and vitamin D_3_ levels.

Described by Reaven in 1988, MetS is characterized by the coexistence of carbohydrate metabolism disorders, lipid metabolism disorders, and hypertension underlain by insulin resistance and obesity [17]. The study of Chacko et al. (2011) does not support the hypothesis of the connection between 25(OH)D levels and LDL, HDL, and glucose levels [63]. In a study of Korean postmenopausal women, on the other hand, low serum vitamin D_3_ levels were accompanied by MetS or some of its parameters (especially hypertriglicerides and hypertension) [64]. In another study, based on the data of the 2008–2010 Korean National Health and Nutrition Examination Surveys (KNHANES), the authors did not note a significant relationship between serum 25(OH)D levels and the incidence of MetS in postmenopausal women [65]. Our study also did not provide evidence for statistically significant correlations between selected metabolic parameters, blood pressure, and vitamin D_3_ levels.

One of the diagnostic criteria for MetS is android obesity, since this is the main factor responsible for the development of insulin resistance. As a result of the disturbed balance between proinflammatory and anti-inflammatory cytokines produced in adipose tissue (and elsewhere), a large amount of visceral adipose tissue contributes to chronic inflammation (so-called metabolic inflammation) [17]. According to Roth et al. (2011), vitamin D levels are associated with the metabolism of adipose tissue. Researchers emphasize that the development of insulin resistance and other MetS components in obese people correlates with vitamin D deficiency [66]. Cheng et al. (2010) also assert that a larger amount of adipose tissue is associated with greater vitamin D deficiency. Furthermore, the negative correlation between adipose tissue levels and the level of 25(OH)D is stronger in people with android obesity [43]. In our study, the vast majority of the women (75.63%) had android obesity and nearly half of them had vitamin 25(OH)D deficiency. Nevertheless, the analysis did not demonstrate a correlation between these two.

A European multicenter study, the Survey in Europe on Nutrition and the Elderly, a Concerted Action (SENECA), involving elderly people from 11 European countries, showed that the lowest vitamin D levels were found in Italy and Spain, and the highest in Norway [25]. High vitamin D_3_ levels in Scandinavian countries are ascribed to a diet based largely on fish and fish fat. Low vitamin D levels in sunny countries (such as Italy and Spain), on the other hand, are explained by avoidance of exposure to sunlight by the studied women. Low vitamin levels in Middle Eastern countries (Turkey, Jordan, Iran, etc.) are attributed to dress codes for women that require them to cover the whole body. Vitamin 25(OH)D deficiency is also related to age. The synthesis of the biologically active form of vitamin D decreases with age, which is often accompanied by chronic renal disease. Additionally, people of advanced age tend to avoid exposure to sunlight [25]. Our study confirmed the correlation between age and vitamin D_3_ but, surprisingly, higher levels of vitamin D_3_ were observed in older women.

Menopause is a physiological phenomenon that typically occurs between 45 and 55 years of age. In Poland, the mean age of menopause is 50 years. This stage of life is characterized by the reduced production of hormones in the ovaries. Lower estrogen levels contribute to lower vitamin D-binding protein (DBP), and consequently lower 25(OH)D levels in blood. This deficiency of 25(OH)D can negatively affect bodily functions, resulting in various disorders (especially in women of postmenopausal age). As stated by Kołodziejczyk et al. (2017), women at this age more often undergo laboratory analysis to determine 25(OH)D levels. Those authors found that postmenopausal women diagnosed with MetS had lower levels of this vitamin than their premenopausal counterparts [67]. In our investigation, the study sample consisted of women with a diagnosis of MetS, over half of whom were postmenopausal. However, our results were different, andvitamin D_3_ deficiency was observed in the premenopausal women.

Vitamin D_3_ exerts an influence on various physiological functions, such as reproduction, sex hormone synthesis, normalization of the menstrual cycle, immune system response, and metabolic pathways [68]. Hence, the need to continue research in this field to expand our knowledge of the pleiotropic effects of vitamin D_3_ seems indisputable.

## 6. Conclusions

This study found the following:

(1) There were no statistically significant relationships between vitamin D_3_ levels and MetS parameters, namely the level of *triglycerides*, *the levels of* LDL, HDL, and total cholesterol, as well as systolic and diastolic blood pressure (SBP and DBP). Vitamin D deficiency was only observed in the women with abdominal obesity.
(2) Low vitamin D_3_ levels were typical of perimenopausal women. Age was a variable correlating with vitamin D.
(3) The presence of menstrual cycles was an important contributor to vitamin D levels. Vitamin D deficiency was significantly more common in the menstruating women.

## Figures and Tables

**Table 1:** Analysis of vitamin D_3_ levels with regard to Body mass index (BMI).

BMI	Vitamin D_3_												
	General Values					*p* *	Deficiency		Optimal Levels		Elevated Levels		*p* **
	*n*	%	M ± SD	Min–Max	Me		*n*	%	*n*	%	*n*	%	
Normal weight	26	21.84	20.27 ± 17.97	0–57.86	13.55	0.092	18	69.23	5	19.23	3	11.54	0.291
Overweight	40	33.61	32.39 ± 29.81	0–133.5	24.45		23	57.50	10	25.00	7	17.50	
Obesity	31	26.05	32.29 ± 24.75	0–85.5	33.09		14	45.16	8	25.81	9	29.03	
Second- or third-degree obesity	22	18.48	39.01 ± 30.31	0–136.4	36.02		8	36.36	9	40.91	5	22.73	

M: mean; SD: standard deviation; Min: minimum; Max: maximum; Me: median; * Lack of normal distribution in the groups; nonparametric analysis; Kruskal–Wallis test; ** Fisher’s exact test (low expected values in the table).

**Table 2:** Analysis of vitamin D_3_ levels with regard to waist circumference.

Waist Circumference	Vitamin D_3_												
	General Values					*p* *	Deficiency		Optimal Levels		Elevated Levels		*p* **
	*n*	%	M ± SD	Min–Max	Me		*n*	%	*n*	%	*n*	%	
Android obesity	90	75.63	33.57 ± 28.19	0–136.4	31.26	0.067	44	48.89	26	28.89	20	22.22	0.291
Lack of obesity	29	24.36	22.79 ± 20.41	0–76.75	14.1		19	65.52	6	20.69	4	13.79	

M: mean; SD: standard deviation; Min: minimum; Max: maximum; Me: median; * Lack of normal distribution in the groups; nonparametric analysis; Kruskal–Wallis test; ** Chi-square test.

**Table 3:** The relationship between vitamin D_3_ and selected metabolic parameters.

Parameter	Correlation with Vitamin D_3_			
	Correlation Coefficient *	*p*	Direction of the Relationship	Strength of the Relationship
SBP [mmHg]	0.056	0.547	---	---
DBP [mmHg]	−0.075	0.416	---	---
Total cholesterol [mg/dL]	−0.01	0.916	---	---
LDL cholesterol [mg/dL]	−0.075	0.42	---	---
HDL cholesterol [mg/dL]	0.095	0.305	---	---
Triglycerides [mg/dL]	0.036	0.701	---	---

* Lack of normal distribution of at least one of the correlated variables; nonparametric analysis; Spearman’s correlation coefficient; *p*: significance level.

**Table 4:** Analysis of the correlation between the women’s age and vitamin D_3_ levels.

Variables	Correlation Coefficient *	*p*	Direction of the Relationship	Strength of the Relationship
Age and vitamin D_3_ levels	0.298	0.001	both variables move in the same direction (positive correlation)	very weak

* Lack of normal distribution of at least one of the correlated variables; nonparametric analysis; Spearman’s correlation coefficient; *p*: significance level.

**Table 5:** The influence of age on the risk of vitamin D_3_ deficiency and the risk of elevated vitamin D_3_ levels.

Parameter	OR	95% CI		*p* *
Risk of vitamin D_3_ deficiency	0.933	0.888	0.981	0.006
Risk of elevated vitamin D_3_ levels	1.131	1.048	1.22	0.002

OR: odds ratio; 95% CI: a 95% confidence interval; *p* *: univariate logistic regression analysis.

**Table 6:** Analysis of vitamin D_3_ levels with regard to the women’s menstrual cycle.

Menstruation	Vitamin D_3_												
	General Values					*p* *	Deficiency		Optimal Levels		Elevated Levels		*p* **
	*n*	%	M ± SD	Min–Max	Me		*n*	%	*n*	%	*n*	%	
Yes	43	36.13	24.30 ± 26.45	0–133.5	15.5	0.015	28	65.12	12	27.91	3	6.98	0.01
No	67	56.30	35.34 ± 27.85	0–136.4	35.02		31	46.27	15	22.39	21	31.34	

M: mean; SD: standard deviation; Min: minimum; Max: maximum; Me: median; * Lack of normal distribution in the groups, nonparametric analysis, Mann-Whitney U test; ** chi-square test.
